# Association between keratoconus, ocular allergy, and sleeping behavior

**DOI:** 10.5935/0004-2749.20210003

**Published:** 2025-02-02

**Authors:** Rodrigo Teixeira Santos, Bernardo Kaplan Moscovici, Flávio Eduardo Hirai, Cláudia Maria Francesconi Benício, Eliane Mayumi Nakano, Walton Nosé

**Affiliations:** 1 Department of Ophthalmology and Visual Sciences, Escola Paulista de Medicina, Universidade Federal de São Paulo, São Paulo, SP, Brazil

**Keywords:** Keratoconus, Hypersensitivity, Sleep/physiology, Rhinitis, allergic, Cornea, Tomography, Ceratocone, Hipersensibilidade, Sono/fisiologia, Ri nite alérgica, Córnea, Tomografia

## Abstract

**Purpose:**

To compare the severity and laterality of keratoconus according to allergic rhinitis, scratching and sleeping habits, and manual dexterity.

**Methods:**

Objective assessments regarding allergic rhinitis, eye itching, and sleeping position among patients with keratoconus (diagnosed based on corneal tomography) were conducted. Diagnostic criteria and classification were based on the Amsler-Krumeich classification.

**Results:**

Ocular pruritus was reported by 29 of 34 participants (85.29%). Eighteen participants (62.07%) reported equal scratching of both eyes, six (20.69%) more on the right eye, and five (17.24%) more on the left eye. Comparison of the main sleeping position and the eye with more severe presentation of the disease using Fisher’s exact test revealed some correlations (0.567 and 0.568 in the right and left eye, respectively). However, these correlations were not statistically significant.

**Conclusions:**

The association between higher keratometry values and sleeping position appears to be more significant than that reported between keratometry and itching, or manual dexterity.

## Abstract (pt)

**Objetivo:**

Comparar a gravidade e a lateralidade do ceratocone de acordo com a rinite alérgica, os hábitos de coçar e dormir e a destreza manual.

**Métodos:**

Foram realizadas questões objetivas sobre rinite alérgica, prurido ocular e posiç ão do sono em pacientes com ceratocone, diagnosticados com base na tomografia corneana. Esses exames foram analisados e classificados de acordo com a classificação de Amsler-Krumeich.

**Resultados:**

O prurido ocular foi referido por 29 (85,29%) dos 34 voluntários. Dezoito sujeitos (62,07%) relataram coçar ambos os olhos igualmente, 6 (20,69%) mais no olho direito e 5 (17,24%) mais no olho esquerdo. Comparando-se a posição de dormir principal e o olhos com apresentação mais grave da doença, foi encontrada alguma relação baseada no teste exato de Fisher (0,567 no olho direito e 0,568 no olho esquerdo), embora nenhuma comparação parecesse estatisticamente significante.

**Conclusões:**

A associação entre maiores valores de ceratometria e posição do sono parece ser mais importante do que entre ceratometria e prurido ou destreza manual.

## INTRODUCTION

Keratoconus is the most common ectasic corneal disease. It is a noninflammatory disease characterized by a focal thinning of the cornea with increased corneal curvature due to the reduced biomechanical strength of the corneal collagen fibers. This condition ultimately leads to decreased visual acuity. Keratoconus is a progressive disease, especially in the first decade of life when the cornea exhibits less rigidity^(1-6)^. Although asymmetric, the disease is bilateral^(7)^. In a review of genetic studies, the majority of keratoconus in families present an autosomal-dominant inheritance pattern with a known genomic loci^(1)^.

The prevalence of keratoconus in the general po pulation is approximately 1 in 2,000 individuals (0.05%)^(7)^. Its etiology is multifactorial, combining environmental, genetic, and behavioral factors. Of note, its distribution differs worldwide; countries with less sun exposure have a lower prevalence than those with greater exposure^(1-6)^. However, it is thought that the higher prevalence may be attributed to ethnic and behavioral characteristics rather than direct sun exposure. However, the association between atopy and the act of eye rubbing has been established as a trigger for the disease development and progression. In numerous studies, half of the participants with keratoconus reported that they rub their eyes, although these findings are variable in the literature. Other influential factors are the frequency and intensity of eye rubbing^(8-11)^.

Microtrauma caused in the epithelium through the friction of the corneas generates high levels of matrix metalloproteinases (MMPs) (i.e., MMP-1 and MMP-13) and inflammatory mediators, including interleukin-6 (IL-6) and tumor necrosis factor-5 (TNF-5). The release of these factors is part of the processes that lead to the manifestation and progression of the disease. These processes include apoptosis of keratocytes as a result of the increased levels of IL-1 with subsequent loss of stromal volume^(5,8)^.

Other factors, such as ethnicity, geographic location (possible exposure to ultraviolet radiation), and socioeconomic factors, are controversial^(1-4)^. Associations between keratoconus and other conditions, such as floppy eyelid syndrome (FES) and obstructive sleep apnea, were described in some studies presented at the Association for Research in Vision and Ophthalmology Meeting in 2012^(12,13)^. The strongest association was found with FES compared with sleep apnea. This finding could be due to the fact that the compression of eyes on the pillow may be sufficiently strong, leading to changes in the palpebral tarsus structure and keratoconus development^(12,13)^.

The purpose of this study was to verify the relationship between the severity of keratoconus, eye rubbing, sleeping habits, manual dexterity and allergic rhinitis.

## METHODS

### Study design

This was a non-interventional comparative case series, conducted between May 2015 and July 2016 in the Department of Ophthalmology and Visual Sciences of the Federal University of São Paulo (São Paulo, Brazil). The study included individuals with keratoconus who were candidates for intrastromal corneal ring surgery. The study was approved by the Institutional Review Board (number: 1.636.206) and followed the tenets of the Declaration of Helsinki. All patients provided written informed consent.

### Participants

Individuals with documented keratoconus were enrolled. The exclusion criteria were any previous ocular surgery, pregnancy or breastfeeding, presence of corneal degenerations (except keratoconus), and other ocular diseases that could influence the ocular examination. Individuals with major comorbidities, such as diabetes and collagen-related diseases, were also excluded.

The diagnosis of keratoconus was based on corneal tomography mapping (mean simulated keratometry >45.2 diopter (D), central corneal power >47.2 D, or inferior-superior asymmetry >1.4 D) using a Pentacam (OCULUS Optikgerate GmbH, Wetzlar, Germany).

### Measurements

An examiner asked the participants objective questions regarding allergic rhinitis, eye rubbing, manual dexterity, and sleeping position. Another examiner collected the corneal tomography data of both eyes. We used the simulated central keratometry data of each eye, maximum keratometry (Kmax) in each eye, and Amsler-Krumeich classification for the analysis.

### Statistical analysis

All data were collected and presented in contingency tables. Means ± standard deviation and frequencies (proportions) were presented for continuous and categorical variables, respectively. For categorical variables, between-group analysis was conducted at each follow-up visit using Fisher’s exact test. Continuous variables were compared using the Mann-Whitney U test and the Pearson coefficient was used for correlation analysis. Statistical analysis was conducted using the Stata v.14 software (College Station, TX, USA), and *p*-values <0.05 indicated statistical significance.

## RESULTS

A total of 34 individuals were evaluated, of whom 14 were females (41.2%) and 20 were males (58.8%). The mean ± standard deviation age was 26.5 ± 7.5 years (range: 17-49 years; median: 25.5 years). There was a weak negative correlation between age and Kmax values of the right eye (Pearson correlation coefficient: -0.321) and left eye (-0.189). There were no associations between age and the preferred eye for rubbing and preferred side for sleeping.

Table 1 presents the main characteristics of the study participants. The presence of allergic rhinitis and ocular itching was reported in 26 (76.5%) and 29 (85.3%) participants, respectively. All participants with allergic rhinitis reported ocular pruritus. Regarding the preferred rubbing eye, the distribution among the right, left, and both sides was similar. Almost half of the participants were unsure of their preferred eye. When asked, 14 participants (41.2%) responded that they sleep most of the time on the right side, and 25 (73.5%) confirmed that they wake up in a position different from their initial sleeping position.

**Table 1:** Characteristics of the study participants (*N*=34)

Allergic rhinitis	
Yes	26 (76.5)
No	8 (23.5)
Ocular itching	
Yes	29 (85.3)
No	5(14.7)
Eye rubbing, preferred eye	
Right eye	5(14.7)
Left eye	6(17.6)
Both	5(14.7)
Not sure	18(53.0)
Sleeping position, preferred side	
Right	14 (41.2)
Left	7 (20.6)
Other position	13 (38.2)
Change of position during sleep	
Yes	25 (73.5)
No	9 (26.5)

Data are expressed as frequency (proportion).

In this study, 19 participants (55.9%) had the worst degree of keratoconus in the right eye; 28 right eyes (82.3) versus 21 left eyes (65.6%) were classified as degree 4 (Table 2).

**Table 2:** Amsler-Krumeich classification of each eye of study participants

Amsler-Krumeich classification	Right eye (*N*=34)	Left eye (*N*=32)
0	1 (2.9)	2 (6.3)
1	1 (2.9)	3 (9.4)
2	2 (5.9)	5 (15.6)
3	2 (5.9)	1 (3.1)
4	28 (82.4)	21 (65.6)

Data are expressed as frequency (proportion).

We compared the Kmax values of the right and left eyes between groups to investigate the influence of three characteristics (i.e., preferred rubbing eye, preferred sleeping side, and hand dominancy). We hypothesized that the keratometric values of these characteristics would be higher in accordance with the preferred side (Table 3). Although none of the differences were statistically significant, we observed a tendency for Kmax values to be higher in accordance with the preferred side for sleeping.

**Table 3:** Comparison of maximum keratometry (Kmax) of right and left eyes according to the characteristics of the participants (*N*=34)

	Rubbing		p-value
	Prefer right eye	Prefer left eye	
Kmax, OD	59.8 ± 10.4	59.5 ± 7.6	0.952
Kmax, OS	56.8 ± 5.2	54.4 ± 7.0	0.552
	**Sleeping side**		
	**Prefer right side**	**Prefer left side**	**p-value**
Kmax, OD	63.6 ± 5.6	58.6 ± 7.7	0.103
Kmax, OS	57.7 ± 7.7	61.2 ± 5.8	0.329
	**Dominant hand**		
	**Right**	**Left**	**p-value**
Kmax, OD	61.8 ± 5.8	59.9 ± 5.9	0.598
Kmax, OS	58.4 ± 6.3	58.0 ± 10.7	0.921

Data are expressed as mean ± standard deviation. OD= oculus dexter; OS= oculus sinister.

## DISCUSSION

The analysis of these data indicates the tendency between higher keratometric values and preferred side for sleeping; however, we did not observed the tendency between higher keratometric values and preferred eye for rubbing. Other studies found a relationship between keratoconus and ocular itching. We could not find a statistically significant relationship between eye rubbing and increased keratometry. However, in our daily practice, we observed that eye rubbing is strongly correlated with keratoconus.

In an investigation including only individuals with clinical unilateral keratoconus, the authors observed a tendency for patients to sleep on the same side with the eye that is most severely affected or the eye with a progressing disease. Some of these patients slept with their hand or fist positioned directly against their eyelid and were more likely to hug their pillow in a manner that caused compression around their eyes^(14)^.

A study investigating the sleeping position through lisamine green staining demonstrated a difference between back sleeping and left-side sleeping (analysis of variance, p=0.005). The Ocular Surface Disease Index score was also increased in patients who slept on their right or left side (36.4 and 34.1, respectively) as oppo-Sleeping position, preferred side sed to those who sleep on their back (26.7) (p=0.05).

There was no statistically significant correlation between the sleeping position and degree of meibomian gland dysfunction^(15)^. In a Japanese study, poor sleep quality was associated with dry eye disease, especially dry eye symptoms^(16,17)^.

Normal eyelid closure has also been linked to the development of several ocular surface disorders. Sleep disorders are common; obstructive sleep apnea (the most common disorder) is associated with a number of serious systemic diseases and several eye disorders, including FES, optic neuropathy, glaucoma, anterior ischemic optic neuropathy, and papilledema secondary to increased intracranial pressure. At the onset of sleep, the lids are closed, and the position of the globes, as judged by the position of the cornea behind the closed lids, is generally elevated. Lagophthalmos may cause corneal exposure that results in pain and foreign body sensation upon waking^(18,19)^. These effects could induce eye rubbing.

In another study, Bawazeer et al. found an association between keratoconus and atopy, as well as eye rubbing and family history of keratoconus. However, in the multivariate analysis, only eye rubbing remained a significant risk factor for the development of keratoconus (odds ratio = 6.31)^(20)^. These findings support the hypothesis that eye rubbing is the most significant cause of keratoconus. Atopy may contribute to keratoconus, most probably *via* eye rubbing due to itching. In that study, there were no other variables significantly associated with the etiology of keratoconus^(20)^.

In a study investigating the association between corneal curvature and eye itching severity, it was verified that the most curved corneas were present in the eyes with more frequent and intense pruritus^(21)^. A series of cases also verified the asymmetric expression of keratoconus and found that individuals habitually rubbed the most affected eye^(21-23)^.

The technique used by many individuals with keratoconus to rub their eyes is usually different from that used by those without keratoconus^(24)^. Individuals with keratoconus tend to use more often the fingertips or even the distal interphalangeal joints to vigorously rub their eyes^(14)^.

An Australian study, involving 64 participants wearing contact lenses (half with keratoconus and half without corneal ectasia), found a significant increase in ocular pruritus after contact lens removal in the keratoconus group. The mean duration of pruritus was significantly longer in the group with keratoconus than without ectasia (27.7 vs. 14.4 s, respectively)^(10)^.

Recently, an increasing body of evidence suggests that inflammatory pathways may play a significant role in the development of keratoconus. Several studies have investigated the role of proteolytic enzymes, such as MMPs, in keratoconus. MMPs are involved in the degradation of the extracellular matrix or activation of cellular apoptosis^(25)^. In the human cornea, MMPs are secreted by epithelial cells, stromal cells, and neutrophils^(26)^. In keratoconus, the cornea expresses increased levels of MMP-117 and MMP-13^(27)^. The tear analysis in keratoconus has revealed increased levels of MMP-1, MMP-3, MMP-7, and MMP-13^(28)^. Increased gelatinolytic and collagenolytic activities have also been reported in the cornea^(29-31)^ and tear film of patients with keratoconus^(28)^.

MMP-9 activity is also high in the tear fluid of patients with keratoconus. Hence, the increase in MMP-9 levels is correlated with corneal thinning, probably as a result of stromal collagen degradation^(28)^. In addition, TNF-α disrupts the barrier function of corneal epithelial cells. The type of cell from which the production of TNF-α in keratoconus originates remains unknown. However, TNF-α can be produced by a variety of cells, including all three major cell types in the cornea: the corneal epithelium, stromal keratocytes, and endothelial cells. Perhaps, corneal damage induced by environmental factors causes the production of TNF-α. For example, eye rubbing and dry eye disease are major risk factors for developing KC and are associated with the induction of TNF-α production by corneal epithelial cells^(32-34)^.

The total tear protein level was significantly reduced in individuals with keratoconus (4.1 ± 0.9 mg/ml) compared with healthy individuals (6.7 ± 1.4 mg/ml) (*p*<0.0001) or those who had undergone corneal collagen cross-linking (5.7 ± 2.3 mg/ml) (*p*<0.005)^(28)^. In a study of healthy participants, there was an increase in the concentration of MMP-13 and inflammatory molecules IL-6 and TNF-α after 60 s of ocular pruritus^(8)^.

The exact mechanism through which keratoconus worsens due to the mechanical trauma caused by eye rubbing or scratching has not yet been elucidated. It has been proposed that IL-1 plays a major role in this process. Wilson et al. suggested that the increased expression of the IL-1 receptor sensitizes the keratocytes to IL-1 released from the epithelium or endothelium. This effect causes loss of keratocytes through apoptosis and a decrease in stromal mass over time. This hypothesis supports that the occurrence of keratoconus is related to eye rubbing, use of contact lenses, and atopy, presuming that epithelial microtrauma leads to an increased release of IL-1 from the epithelium^(35,36)^.

Our study had some limitations. First, this study may have not been statistically powered to detect associations due to the small sample size. Second, characteristics, such as eye rubbing and sleeping side, were reported by the participants without a more objective assessment. However, our findings support the importance of allergy control and eye trauma avoidance among those at risk of developing keratoconus.

In conclusion, our study revealed a tendency of the eyes with most advanced degrees of keratoconus to be associated with allergy, eye rubbing, and preferred sleeping side.

## References

[1] Gordon-Shaag A, Millodot M, Shneor E, Liu Y. (2015). The Genetic and environmental factors for
keratoconus. Biomed Res Int.

[2] Gomes JA, Rapuano CJ, Belin MW, Ambrósio Jr R, Group of Panelists for the Global Delphi Panel of Keratoconus and
Ectatic Diseases (2015). Global consensus on keratoconus diagnosis. Cornea.

[3] Galvis V, Sherwin T, Tello A, Merayo J, Barrera R, Acera A. (2015). Keratoconus: an inflammatory disorder?. Eye (Lond).

[4] Vazirani J, Basu S. (2013). Keratoconus: current perspectives. Clin Ophthalmol.

[5] Wisse RP, Kuiper JJ, Gans R, Imhof S, Radstake TR, Van der Lelij A. (2015). Cytokine expression in keratoconus and its corneal
microenvironment: a systematic review. Ocul Surf.

[6] Gordon-Shaag A, Millodot M, Kaiserman I, Sela T, Barnett Itzhaki G, Zerbib Y (2015). Risk factors for keratoconus in Israel: a case-control
study. Ophthalmic Physiol Opt.

[7] Rabinowitz YS. (1998). Keratoconus. Surv Ophthalmol.

[8] Balasubramanian SA, Pye DC, Willcox MD. (2013). Effects of eye rubbing on the levels of protease, protease
activity and cytokines in tears: relevance in keratoconus. Clin Exp Optom.

[9] Panahi-Bazaz MR, Sharifipour F, Moghaddasi A. (2014). Bilateral keratoconus and corneal hydrops associated with eye
rubbing in a 7-year-old girl. J Ophthalmic Vis Res.

[10] McMonnies CW. (2016). Eye rubbing type and prevalence including contact lens
‘removal-relief’ rubbing. Clin Exp Optom.

[11] McMonnies CW, Korb DR, Blackie CA. (2012). The role of heat in rubbing and massage-related corneal
deformation. Cont Lens Anterior Eye.

[12] Gencer B, Ozgurhan EB, Kara S, Tufan HA, Arikan S, Bozkurt E (2014). Obesity and obstructive sleep apnea in patients with keratoconus
in a turkish population. Cornea.

[13] Pedrotti E, Demasi CL, Fasolo A, Bonacci E, Brighenti T, Gennaro N (2018). Obstructive sleep apnea assessed by overnight polysomnography in
patients with keratoconus. Cornea.

[14] Carlson AN. (2009). Keratoconus: time to rewrite the textbooks. Rev Oph-thalmol.

[15] Alevi D, Perry HD, Wedel A, Rosenberg E, Alevi LB, Donnenfeld ED. (2017). Effect of sleep position on the ocular surface. Cornea.

[16] Kawashima M, Uchino M, Yokoi N, Uchino Y, Dogru M, Komuro A (2016). The association of sleep quality with dry eye disease: the Osaka
study. Clin Ophthalmol.

[17] McNab AA. (2005). The eye and sleep. Clin Exp Ophthalmol.

[18] Lyons CJ, McNab AA. (1990). Symptomatic nocturnal lagophthalmos. Aust NZ J Ophthalmol.

[19] Mueller FO. (1967). Lagophthalmos during sleep. Brit J Ophthalmol.

[20] Bawazeer AM, Hodge WG, Lorimer B. (2000). Atopy and keratoconus: a multivariate analysis. Br J Ophthalmol.

[21] Zadnik K, Steger-May K, Fink BA, Joslin CE, Nichols JJ, Rosenstiel CE, Tyler JA, Yu JA, Raasch TW, Schechtman KB, CLEK Study Group (2002). Collaborative Longitudinal Evaluation of Keratoconus. Between-eye
asymmetry in keratoconus. Cornea.

[22] Jafri B, Lichter H, Stulting RD. (2004). Asymmetric keratoconus attributed to eye rubbing. Cornea.

[23] Koenig SB. (2008). Bilateral recurrent self-induced keratoconus. Eye Con-tact Lens.

[24] De Benedetto A, Agnihothri R, McGirt LY, Bankova LG, Beck LA. (2009). Atopic dermatitis: a disease caused by innate immune
defects?. J Invest Dermatol.

[25] Ollivier FJ, Gilger BC, Barrie KP, Kallberg ME, Plummer CE, O’Reilly S (2007). Proteinases of the cornea and preocular tear film. Vet Ophthalmol.

[26] Fini ME, Cook JR, Mohan R. (1998). Proteolytic mechanisms in corneal ul-ceration and
repair. Arch Dermatol Res.

[27] Mackiewicz Z, Maatta M, Stenman M, Konttinen L, Tervo T, Konttinen YT. (2006). Collagenolytic proteinases in keratoconus. Cornea.

[28] Balasubramanian SA, Mohan S, Pye DC, Willcox MD. (2012). Proteases, proteolysis and inflammatory molecules in the tears of
people with keratoconus. Acta Ophthalmol.

[29] Kao WW, Vergnes JP, Ebert J, Sundar-Raj CV, Brown SI. (1982). Increased collagenase and gelatinase activities in
keratoconus. Biochem Biophys Res Commun.

[30] Brown D, Chwa MM, Opbroek A, Kenney MC. (1993). Keratoconus cor-neas: increased gelatinolytic activity appears
after modification of inhibitors. Curr Eye Res.

[31] Becker J, Salla S, Dohmen U, Redbrake C, Reim M. (1995). Explorative study of interleukin levels in the human
cornea. Graefes Arch Clin Exp Ophthalmol.

[32] Balasubramanian SA, Pye DC, Willcox MD. (2013). Effects of eye rubbing on the levels of protease, protease
activity and cytokines in tears: relevance in keratoconus. Clin Exp Optom.

[33] Meloni M, De Servi B, Marasco D, Del Prete S. (2011). Molecular mecha-nism of ocular surface damage: application to an
in vitro dry eye model on human corneal epithelium. Mol Vis.

[34] Hong JW, Liu JJ, Lee JS, Mohan RR, Woods DJ, He YG (2001). Proinflammatory chemokine induction in keratocytes and
inflammatory cell infiltration into the cornea. Invest Ophthalmol Vis Sci.

[35] Bron AJ, Rabinowitz YS. (1996). Corneal dystrophies and keratoconus. Curr Opin Ophthalmol.

[36] Shetty R, Ghosh A, Lim RR, Subramani M, Mihir K, Reshma AR (2015). Elevated expression of matrix metalloproteinase-9 and
inflammatory cytokines in keratoconus patients is inhibited by cyclosporine
A. Invest Ophthalmol Vis Sci.

